# Quantifying Phylogenetic Beta Diversity: Distinguishing between ‘True’ Turnover of Lineages and Phylogenetic Diversity Gradients

**DOI:** 10.1371/journal.pone.0042760

**Published:** 2012-08-17

**Authors:** Fabien Leprieur, Camille Albouy, Julien De Bortoli, Peter F. Cowman, David R. Bellwood, David Mouillot

**Affiliations:** 1 Laboratoire Ecologie des Systèmes Marins Côtiers UMR 5119, Université Montpellier 2, Montpellier, France; 2 Australian Research Council Centre of Excellence for Coral Reef Studies and School of Marine and Tropical Biology, James Cook University, Townsville, Queensland, Australia; University of Akron, United States of America

## Abstract

The evolutionary dissimilarity between communities (phylogenetic beta diversity PBD) has been increasingly explored by ecologists and biogeographers to assess the relative roles of ecological and evolutionary processes in structuring natural communities. Among PBD measures, the PhyloSor and UniFrac indices have been widely used to assess the level of turnover of lineages over geographical and environmental gradients. However, these indices can be considered as ‘broad-sense’ measures of phylogenetic turnover as they incorporate different aspects of differences in evolutionary history between communities that may be attributable to phylogenetic diversity gradients. In the present study, we extend an additive partitioning framework proposed for compositional beta diversity to PBD. Specifically, we decomposed the PhyloSor and UniFrac indices into two separate components accounting for ‘true’ phylogenetic turnover and phylogenetic diversity gradients, respectively. We illustrated the relevance of this framework using simple theoretical and archetypal examples, as well as an empirical study based on coral reef fish communities. Overall, our results suggest that using PhyloSor and UniFrac may greatly over-estimate the level of spatial turnover of lineages if the two compared communities show contrasting levels of phylogenetic diversity. We therefore recommend that future studies use the ‘true’ phylogenetic turnover component of these indices when the studied communities encompass a large phylogenetic diversity gradient.

## Introduction

Phylogenies are increasingly used (i) to understand the origins and histories of species within a community (i.e. alpha diversity), (ii) to assess the relative roles of environmental sorting, competitive exclusion and evolutionary and biogeographical processes in shaping community structure [1]–[3] (iii) to predict the level of ecosystem functioning [4] and the delivery of services [5], and (iv) to guide conservation prioritization [6], [7]. These arguments have recently been extended to phylogenetic beta diversity (PBD hereafter) that measures the phylogenetic dissimilarity among communities [8]–[10]. These authors argue that PBD allows a better understanding of the mechanisms underlying current biodiversity patterns by connecting local processes (e.g. biotic interactions and environmental filtering) with more regional processes, including trait evolution, speciation and dispersal.

To quantify PBD, numerous measures have been proposed [11], and more particularly two indices that derive from the taxonomic-based Sorenson and Jaccard's dissimilarity indices, namely the PhyloSor [8] and UniFrac [12] indices. These two closely related indices belong to a family of phylogenetic diversity-based dissimilarity measures, i.e. based on calculations using branch lengths [13]. For instance, the UniFrac index is expressed as the total branch length unique to each community relative to the total branch length linking all species in both communities and hence measures the proportion of evolutionary history unique to each community [12].

Numerous studies have employed the PhyloSor and UniFrac indices to explore the spatial turnover of lineages over large spatial scales [3], [10], [13]–[15]. However, these indices are fundamentally based on ‘broad-sense’ measures of compositional beta diversity (CBD) that do not adjust for differences in composition attributable to richness gradients [16]. Consequently, the compositional differences arising from differences in species richness (species loss or gain associated with nestedness) cannot be distinguished from differences in species composition that are independent of species richness (‘true’ species turnover that involves species replacement) [17]–[19]. For instance, recent studies showed that using ‘broad-sense’ measures of CBD could make it difficult to tease apart the relative roles of neutral vs. niche-based processes in shaping CBD patterns [20], [21]. This is because spatial turnover of species and nestedness of assemblages are two antithetic phenomena that are caused by different processes [18], [20]. In that context, Baselga [19] proposed an additive partitioning framework that consists in decomposing the pair-wise Sørensen's dissimilarity index into two additive components accounting for (i) ‘true’ turnover of species and (ii) richness differences between nested communities. Recent studies distinguishing between the ‘true’ turnover and nestedness components of CBD provided new insights into the mechanisms that drive CBD at large spatial scales [20]–[24].

As for their taxonomic-based relatives, the PhyloSor and UniFrac indices can be both considered as ‘broad-sense’ measures of phylogenetic turnover (i.e. incorporating differences in evolutionary history between communities attributable to phylogenetic diversity gradients). Recently, Ives & Helmus [25] tackled this issue by proposing a PBD metric that is independent of species richness in communities. However, this PBD metric does not control for differences in phylogenetic diversity between communities.

In the present study, we therefore extended the framework proposed by Baselga [19], [26] to PBD by decomposing the PhyloSor and UniFrac indices into two components accounting for ‘true’ phylogenetic turnover and phylogenetic diversity gradients, respectively. To illustrate this decomposition, we used theoretical and archetypal examples. We also generated a large number of simulated communities from two contrasted types of phylogenetic tree, i.e. using either PDA (proportional-to-distinguishable arrangements) or Yule model. Finally, we used coral reef fish as a biological model to exemplify the relevance of distinguishing between ‘true’ phylogenetic turnover and phylogenetic diversity gradients when analysing large-scale patterns of PBD with marked differences in species richness and phylogenetic diversity.

## Distinguishing between Phylogenetic Diversity Gradients and Spatial Turnover of Lineages: Formulations

Using an additive partitioning framework, Baselga [19] provided two separate components of species turnover and nestedness underlying the total amount of CBD. Specifically, this framework consists in decomposing the pair-wise Sørensen dissimilarity index (β_sor_) into two additive components accounting for pure species turnover (β_sim_) and nestedness (β_sne_) patterns. The Simpson's dissimilarity index (β_sim_) describes species turnover (or species replacement) without the influence of richness gradients [16], [27]–[29]. Using basic operations on fractions, Baselga [19] derived a Nestedness-resultant dissimilarity index (β_sne_) and showed that β_sne_ is simply the difference between β_sor_ and β_sim_ (i.e. β_sor_ = β_sim_+β_sne_). Specifically, β_sne_ reflects the increasing dissimilarity between nested assemblages due to the increasing differences in species richness [19]. These pairwise dissimilarity indices are formulated as:
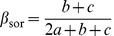
(1)

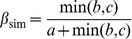
(2)

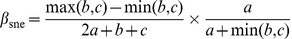
(3)where *a* is the number of species common to both sites, *b* is the number of species that occur in the first site but not in the second and *c* is the number of species that occur in the second site but not in the first [19].

Recently, Baselga [26] proposed a similar decomposition based on the Jaccard's dissimilarity index. The following pairwise dissimilarity indices (formulas 5 and 6) represent, the turnover and nestedness components of the Jaccard's dissimilarity index (β_jac_ = β_jtu_+β_jne_), respectively.
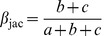
(4)

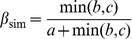
(5)

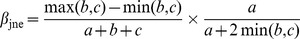
(6)Specifically, β_jtu_ measures the proportion of species that would be replaced between communities if both communities had the same number of species, and hence accounts for species replacement without the influence of richness difference [26]. In contrast, β_jne_ reflects the increasing dissimilarity between nested assemblages due to the increasing differences in species richness. Baselga [26] showed that the results obtained by the closely related Jaccard and Sørensen's dissimilarity indices were roughly equivalent.

Considering two communities *j* and *k* for which biodiversity can be quantified in terms of phylogenetic trees (*T_j_* and *T_k_* are the subset of a rooted regional tree *T*), we can express *a* as the sum of lengths for branches that are shared between communities *j* and *k*, *b* as the sum of lengths for branches that are present in community *j* but not found in assemblage *k*, *c* as the sum of lengths for branches that are present in community *k* but not found in community *j*. We express *b, c* and *a* using the phylogenetic diversity index [30], [31] that can be calculated as the total branch length of a phylogenetic tree *T* that contains all species present in a community. Each branch *t* in the tree *T* has a length of *w_t_*.
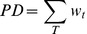
(7)

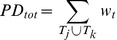
(8)

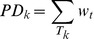
(9)

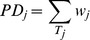
(10)

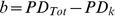
(11)

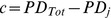
(12)

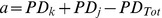
(13)

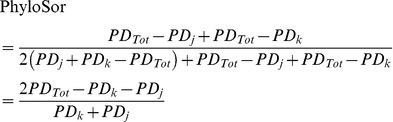
(14)

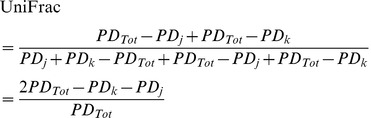
(15)Overall, both the PhyloSor and UniFrac indices range from 0 (the two communities are composed of similar species and hence share the same branches in the rooted phylogenetic tree) to 1 (the two communities are composed of distinct species that share no branch in the rooted phylogenetic tree). The two indices differ only because PhyloSor double weights the branch lengths shared by the two communities (i.e. the denominator of PhyloSor corresponds to the sum of phylogenetic diversity characterizing each community).

Following the formula (2), we obtained the turnover components of the PhyloSor and UniFrac indices, i.e. PhyloSor_Turn_ and UniFrac_Turn_, respectively:
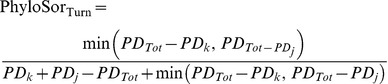
(16)

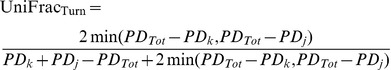
(17)The phylogenetic diversity (hereafter PD) component of PhyloSor is simply the difference between PhyloSor and PhyloSor_Turn_, i.e. PhyloSor_PD_ = PhyloSor – PhyloSor_Turn_. It can be expressed using the formula (3) and by replacing *a*, *b* and *c* by the formula (10), (9) and (8), respectively. Similarly, the PD component of UniFrac is the difference between UniFrac and UniFrac_Turn_, i.e. UniFrac_PD_ = UniFrac – UniFrac_Turn_. It can be expressed using the formula (6) and by replacing *a*, *b* and *c* by the formula (10), (9) and (8), respectively. All the analyses presented in this study were performed using the R statistical and programming environment [32]. The R code required to apply the additive partitioning framework is provided as Supporting Information (File S1 and File S2), together with the community dataset and the phylogenetic tree used to exemplify our approach (File S3 and File S4, respectively).

**Figure 1:**
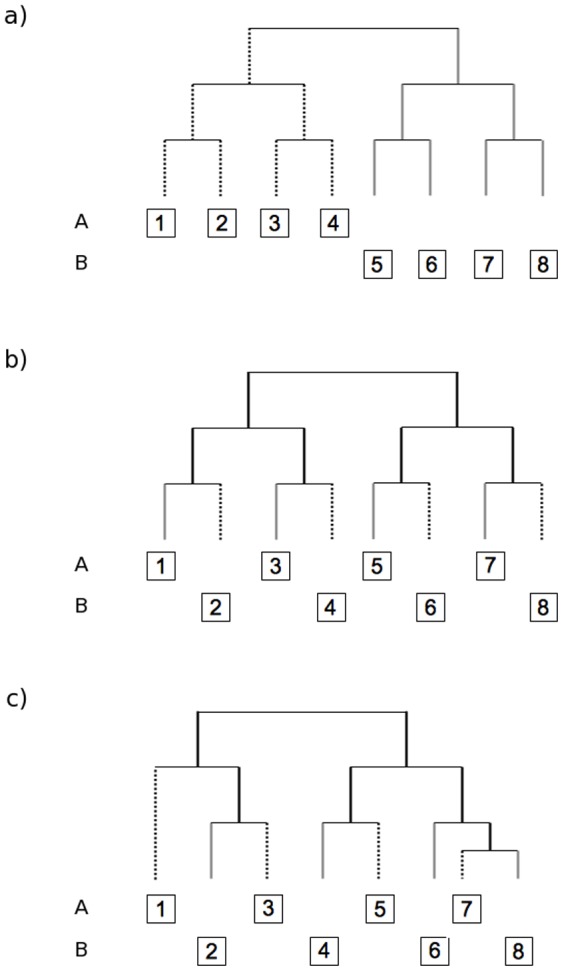
Three simple examples of phylogenetic tree associated to a pair of communities. Each community is composed of four species. All species are scored as present or absent in each example. All branch lengths are set to one, except for the last example (c) for which species 7 and species 8 display a branch length equal to 0.5. The three examples show similar level of compositional beta diversity (i.e. communities have no species in common) but differ in regards to the level of phylogenetic beta diversity (see main text for more details).

As a simple illustration of the proposed decomposition of PBD, the figure 1 presents three different examples. The first two examples (Fig. 1a and 1b, respectively) show two communities (A and B) that have no species in common (β_sor_ = β_sim_ = 1 and β_sne_ = 0). However, communities A and B (example 1, Fig. 1a.) display distantly related species, hence indicating locally phylogenetically clustered communities that have high PBD (PhyloSor = 1, i.e. the two communities compared do not share evolutionary history). In contrast, communities A and B illustrating the example 2 (Fig. 1b) display closely related species, hence indicating locally phylogenetically overdispersed communities that have little PBD (PhyloSor = 0.4, i.e. the two communities share a large amount of evolutionary history). For both examples, PhyloSor = PhySor_Turn_ as PhySor_PD_ = 0. For the first example, the PD component of PBD is zero because the two communities do not share any branch length and also display a similar level of PD (PD_A_ = PD_B_ = 7, see Fig. 1a). For the second example, the PD component of PBD is zero only because the two communities compared display the same level of PD (PD_A_ = PD_B_ = 10, see Fig. 1b). Indeed, if we reconsider the example 2 with two communities having slightly unequal levels of PD (Fig. 1c, PD_A_ = 10 and PD_B_ = 9), the overall level of PBD (PhyloSor = 0.421) is found to be different from the turnover component of PBD (PhySor_Turn_ = 0.388). The difference between the two indices (expressed as PhySor_PD_) quantifies the amount of PBD caused by a difference in PD between the two communities.

**Figure 2:**
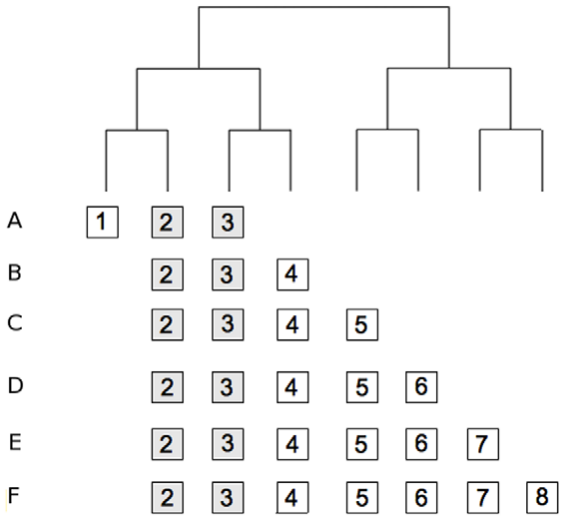
An example of six communities associated to a hypothetical phylogenetic tree. All species are scored as present or absent in each example and all branch lengths are set to one. Phylogenetic beta diversity (PBD) values were computed for several pairs of communities according to the PhyloSor and UniFrac indices and their respective turnover and phylogenetic diversity components (see Table 1 and main text for more details).

Let now consider 6 different communities sharing only two species (species 2 and 3 in Fig. 2). The phylogenetic diversity unique to community A remains constant while the phylogenetic diversity unique to the other communities increases from B to F. Comparisons between the community A and the other communities (B to F, see Table 1) show that the increasing phylogenetic beta diversity (PhyloSor and UniFrac) is entirely caused by an increasing contribution of the PD component (PhySor_PD_ and UniFrac_PD_), while the turnover component remains constant across comparisons (PhySor_Turn_ = 0.166 and UniFrac_Turn_ = 0.286).

**Table 1:** Numerical examples showing the decomposition of the total amount of phylogenetic beta diversity into two components accounting for ‘true’ turnover of lineages and difference in phylogenetic diversity, respectively.

	a	b	c	PhyloSor	PhyloSor_Turn_	PhyloSor_PD_	UniFrac	UniFrac_Turn_	UniFrac_PD_
A-B	5	1	1	0.166	0.166	0	0.286	0.286	0
A-C	5	1	4	0.333	0.166	0.167	0.500	0.286	0.214
A-D	5	1	5	0.375	0.166	0.209	0.545	0.286	0.259
A-E	5	1	7	0.444	0.166	0.278	0.615	0.286	0.329
A-F	5	1	8	0.474	0.166	0.308	0.643	0.286	0.357
B-C	6	0	3	0.2	0	0.2	0.333	0	0.333
B-D	6	0	4	0.25	0	0.25	0.400	0	0.400
B-E	6	0	6	0.333	0	0.333	0.500	0	0.500
B-F	6	0	7	0.368	0	0.368	0.538	0	0.538

The decomposition was based on the PhyloSor and UniFrac indices according to different pairwise comparisons involving the phylogenetic tree presented in Fig. 2. a: branch length shared by the two communities; b and c: branch length unique to the two communities compared, respectively.

It is worth noting that PhyloSor_PD_ = PhyloSor (or UniFrac_PD_ = UniFrac) when the two communities compared are completely nested in regards to their taxonomic composition (see Fig. 2 and Table 1). For instance, the community B has no unique species and hence the branch length unique to community B is zero. Comparisons between the community B and the other communities (C to F, see Table 1) show that the increasing phylogenetic beta diversity (PhyloSor and UniFrac) is entirely caused by an increasing contribution of the PD component while the turnover component remains equal to 0.

Overall, the above examples (Fig. 1 and 2, Table 1) emphasize that PhyloSor_Turn_ and UniFrac_Turn_ are two ‘narrow-sense’ measures of PBD (i.e. ‘true’ measures of phylogenetic turnover) that are independent of total branch length difference between the two compared communities (see Fig. 2 and Table 2). Specifically, PhyloSor_Turn_ and UniFrac_Turn_ measure the relative magnitude of gain and loss of unique lineages between communities that is not attributable to their difference in PD (i.e. phylogenetic turnover expected if the two communities display similar levels of PD). In contrast, PhyloSor_PD_ and UniFrac_PD_ measure the amount of PBD caused by PD differences between phylogenetically nested communities (i.e. communities sharing at least one branch within a rooted phylogeny).

**Table 2:** Results showing the decomposition of PBD of coral reef fish communities into two components accounting for turnover of lineages and differences in phylogenetic diversity, respectively.

	Decomposition based on PhyloSor						Decomposition based on UniFrac					
Pairwise comparisons	PhyloSor	PhyloSor_Turn_	PhyloSor_PD_	SES.PhyloSor	SES.PhyloSor_Turn_	SES.PhyloSor_PD_	UniFrac	UniFrac_Turn_	UniFrac_PD_	SES.UniFrac	SES.UniFrac_Turn_	SES.UniFrac_PD_
Mauritius-Togian	0.44	0.32	0.12	1.07	2.96	−2.32	0.62	0.48	0.14	1.07	2.75	−2.49
Mauritius-Moorea	0.29	0.19	0.10	−0.35	0.61	−1.06	0.45	0.32	0.13	−0.33	0.64	−1.05
Mauritius-Panama	0.74	0.32	0.42	1.16	0.00	0.95	0.85	0.48	0.37	1.15	0.07	0.43
Mauritius-GBR	0.31	0.14	0.17	−1.36	−0.24	−1.03	0.47	0.25	0.22	−1.38	−0.22	−0.79
Mauritius-Vanuatu	0.33	0.26	0.07	−1.38	1.19	−2.80	0.50	0.41	0.09	−1.41	1.19	−2.61
Togian-Moorea	0.35	0.31	0.04	2.10	2.81	−1.38	0.52	0.47	0.05	2.03	2.63	−1.51
Togian-Panama	0.81	0.32	0.49	1.28	1.05	−0.62	0.90	0.49	0.41	1.27	1.07	−0.91
Togian-GBR	0.26	0.25	0.01	2.26	2.64	−0.95	0.41	0.40	0.01	2.19	2.51	−0.98
Togian-Vanuatu	0.30	0.23	0.07	2.96	1.49	1.23	0.47	0.38	0.09	2.82	1.46	0.90
Moorea-Panama	0.80	0.35	0.45	0.94	0.11	0.40	0.89	0.52	0.37	0.94	0.18	0.07
Moorea-GBR	0.23	0.17	0.06	2.12	2.39	−0.53	0.37	0.29	0.08	2.05	2.31	−0.76
Moorea-Vanuatu	0.24	0.21	0.03	1.74	2.05	−0.77	0.38	0.35	0.03	1.70	1.99	−0.86
Panama-GBR	0.82	0.35	0.47	1.14	0.42	0.03	0.91	0.52	0.39	1.14	0.49	−0.29
Panama-Vanuatu	0.79	0.35	0.44	0.40	0.33	−0.21	0.88	0.52	0.36	0.42	0.39	−0.35
GBR-Vanuatu	0.16	0.07	0.09	1.38	−1.60	3.73	0.28	0.13	0.15	1.37	−1.65	3.72

The decomposition was based on PhyloSor and UniFrac. Standardized Effect Size (SES) values were calculated from a null model (i.e. null expectation obtained by randomizing species across the tips of regional phylogenies while holding species richness and CBD constant). GBR: Great Barrier Reef (Australia).

## Theoretical Examples Using Simulated Communities

We simulated pairwise comparisons of communities by taking random values of *a*, *b* and *c* matching components (see formula 1 to 6) from uniform distributions between 1 and 100, where *a* is the number of species common to both communities, *b* is the number of species that occur in the first community but not in the second and *c* is the number of species that occur in the second community but not in the first. The regional species pool is thus composed of 100 species. 10 000 local communities were generated.

For each pairwise comparison, we quantified the corresponding PBD (i.e. using PhyloSor and UniFrac), and we applied the proposed decomposition of PBD. To do so, we simulated the phylogenetic relatedness among species by creating two types of regional phylogenetic trees [33], the former being generated from the PDA (proportional-to-distinguishable arrangements) model and the latter generated from the Yule model (see Fig. S1). Specifically, we aimed at testing the influence of phylogenetic tree structure (i.e. balanced vs. unbalanced trees) on the turnover and PD components of PBD. A phylogenetic tree generated under PDA (proportional-to-distinguishable arrangements) model tends to be more unbalanced than observed phylogenies because all trees with the same number of tips (i.e. species) are equally likely and the majority of potential arrangements are uneven [34], [35]. Reversely, a Yule model tends to produce more balanced phylogenetic trees than empirical ones because it assumes a constant speciation/extinction rate along the tree [36], [37]. Consequently, the Yule model induces a higher degree of phylogenetic similarity between species than PDA model does. The two simulated phylogenetic trees (see supplementary figures S2) were created using the R package “apTreeshape" [38].

**Figure 3:**
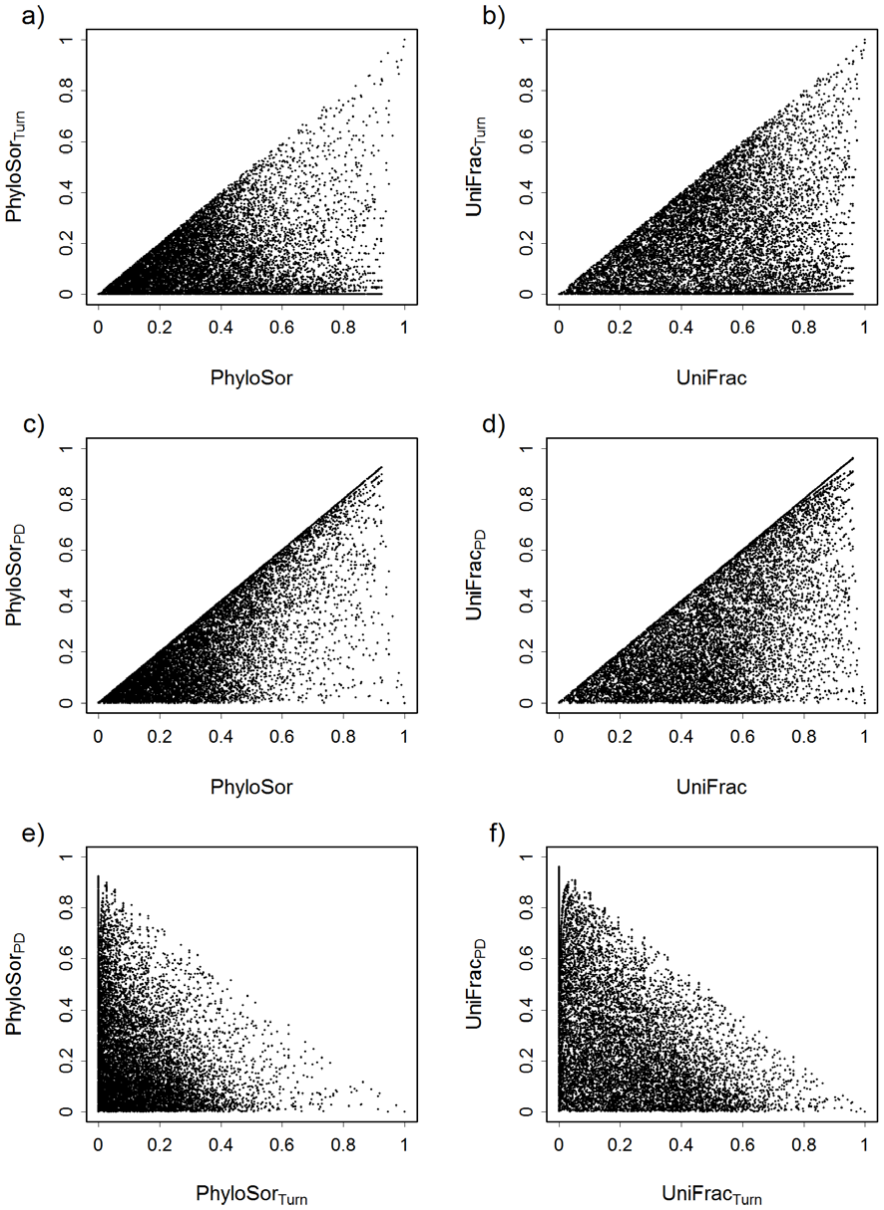
Relationships between phylogenetic beta diversity (PBD) and its turnover and phylogenetic diversity components. These relationships are shown according to the PhyloSor index (a, c, e) and the UniFrac index (b, d, f). Values of PBD were calculated according to simulated communities (see main text for more details).

When considering the PhyloSor index and the PDA phylogenetic tree (see Fig S2), results showed that both the turnover and PD components of PBD displayed a positive triangular relationship with PhyloSor (Fig. 3a and 3c), with an upper bound (first bisectrix) corresponding to the situation where PhyloSor_Turn_ = PhyloSor (Fig. 3a) and PhyloSor_PD_ = PhyloSor (Fig. 3c). PhyloSor_Turn_ = PhyloSor when the two communities compared had the same phylogenetic diversity, whereas PhyloSor_PD_ = PhyloSor when the two communities were completely nested in regards to their taxonomic composition (see also Fig. 2 and Table 1). When comparing PhyloSor_PD_ and PhyloSor_Turn_ together, we found a negative triangular relationship with an upper bound (first bisectrix) corresponding to the cases where PhyloSor = PhyloSor_Turn_+PhyloSor_PD_ = 1. Similar results were obtained when considering UniFrac (Fig. 3b, 3d and 3f). Overall, these relationships based on simulated communities allow verifying the additive property of the proposed decomposition of PBD. Baselga [19] found similar triangular relationships when considering taxonomic beta diversity.

We hypothesized that phylogenetic tree topology (i.e. balance vs. unbalanced trees) may influence the observed patterns of PBD. Results showed high levels of correlation (Pearson's correlation coefficient: *r_p_*>0.95) between PBD values (PhyloSor and UniFrac) obtained using the Yule and PDA phylogenetic trees (Fig. S2). This was also verified when analysing the turnover and PD components of PBD (Fig. S2), while the levels of correlation were found to be lower (Pearson's correlation coefficient: *r_p_*≈0.8). This suggests that the shape of phylogenetic trees may have a weak influence on PBD and its turnover and PD components. However, a deeper work covering a wider panel of tree topologies [39] is needed to fully investigate the influence of phylogenetic tree shapes on PBD measurements.

**Figure 4:**
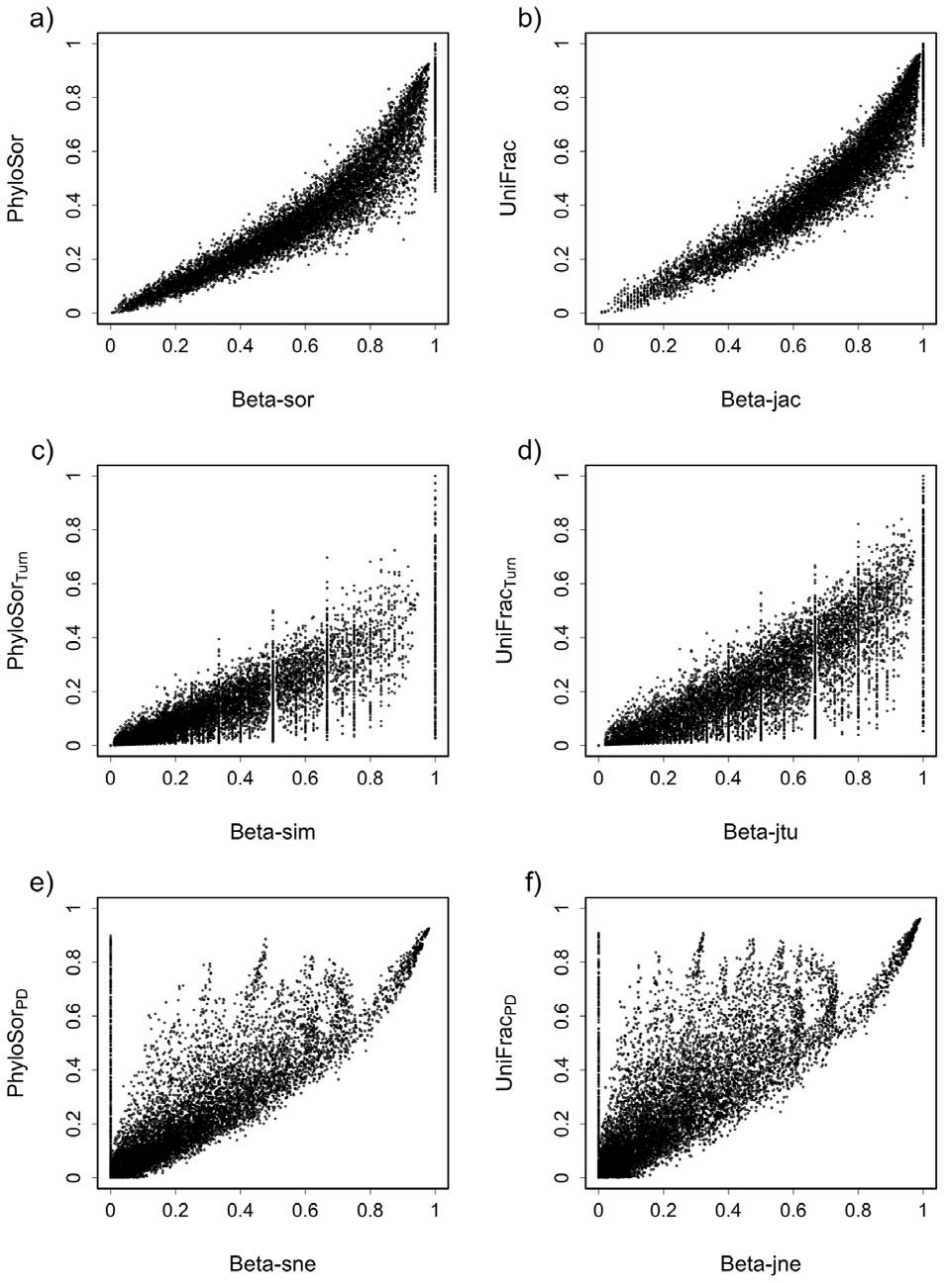
Relationships between phylogenetic beta diversity (PBD) and compositional beta diversity (CBD). These relationships are shown according to the Sorenson's dissimilarity index (a) and the Jaccard's dissimilarity indices (b) and their phylogenetic analogues (PhyloSor and UniFrac). Values of PBD and CBD were calculated according to simulated communities (see main text for more details). The relationship between the turnover components of PBD and CBD are also shown (c and d), as well the relationship between the phylogenetic diversity component of PBD and the nestedness component of CBD (e and f).

Previous empirical studies emphasized that CBD and PBD may be highly correlated [3], [8]. Our simulation-based approach confirmed that both the PhyloSor and UniFrac indices were highly correlated with the Sorensen (Pearson's correlation coefficient: *r_p_* = 0.933, Fig. 4a) and Jaccard dissimilarity (Pearson's correlation coefficient: *r_p_* = 0.942, Fig. 4b) indices, respectively. This was also verified when analysing the turnover and PD components of PBD that showed high levels of correlation with the turnover and nestedness components of CBD (Pearson's correlation coefficient *r_p_* ranging from 0.80 to 0.84, see Fig. 4c,d,e,f). It is worth noting that the phylogenetic diversity component of PBD is not trivially related to the nestedness component of CBD (see Fig. 4a,b). For example, when two communities are non-nested (i.e. β_sne_ or β_jne_ = 0), values of PhyloSor_PD_ and UniFrac_PD_ can be higher than 0. This highlights that PhyloSor_PD_ and UniFrac_PD_ measure the amount of PBD caused by PD differences for both nested and non-nested communities. Overall, these results emphasize that appropriate null models are required to analyze patterns of PBD and underlying processes. For instance, using PhyloSor (or Unifrac) and its turnover and PD components, one can test whether two communities are phylogenetically more or less dissimilar than what is expected given their taxa dissimilarity (CBD). This can be achieved by comparing the phylogenetic dissimilarity of the observed communities to a null expectation obtained by randomizing species across the tips of regional phylogenies while holding species richness and CBD constant [3], [8].

## Empirical Patterns of Phylogenetic Beta Diversity Between Coral Reef Fish Communities

We illustrated the relevance of partitioning PBD into ‘true’ phylogenetic turnover and PD components by exploring patterns of PBD among local communities of coral reef fishes belonging to the family Labridae. The Labridae is a species rich fish family, circa 600 species [40], that is characteristic of coral reef fish faunas around the world [41]. We compiled labrid fish species occurrences for 6 sites distributed along a longitudinal gradient (from the Indian Ocean to the Eastern Pacific passing by the Indo-Australian Archipelago, hereafter IAA, see Table S1). At each site, species occurrences were based on 12×20-min. timed swims (four locations x three habitats; the reef slope, crest and flat), to provide an overview of the local labrid fauna (census details are provided in [42]). This gradient spanned almost the entire Indian and Pacific Oceans, and encompassed the major physical factors that are thought to affect the global distribution of reef fishes [42]. To explore PBD, we used a labrid reef fish phylogeny (108 coral reef fish species recorded from the 6 locations) that was constructed using a genetic algorithm approach based on a maximum likelihood criterion and dated using Bayesian Inference [43].

**Figure 5:**
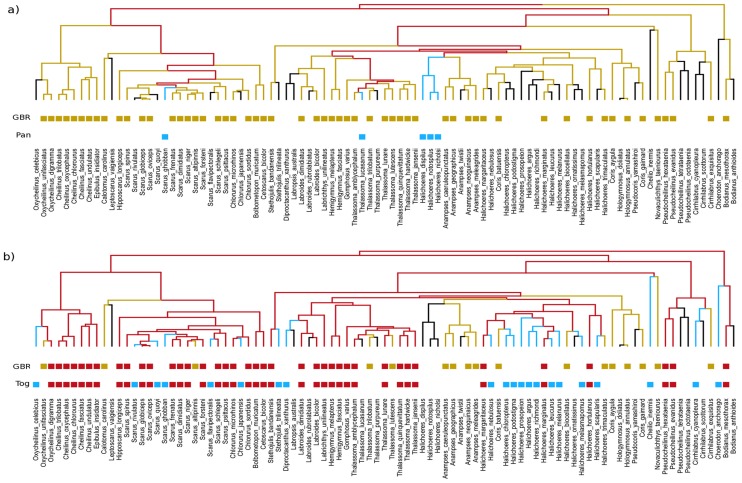
Phylogenetic relationships between two regional pools of labrid reef fish species. Two examples of between-region comparison are presented (a: Great Barrier Reef vs. Panama, and b: Great Barrier Reef vs. Togian). Red color shows branch length shared by the two communities compared. Green and blue colors represent the total branch length unique to each community.

The PhyloSor index showed low levels of PBD between sites (i.e. values of PhyloSor ranging from 0.16 to 0.44, Table 2), except for the pairwise comparisons involving Panama where high levels of PBD were found (e.g. values of PhyloSor ranging from 0.74 to 0.82, see Table 2). Using UniFrac index provided similar results (Table 2). Arguably, one might conclude that high turnover of lineages occurs between Panama (East Pacific) and the other sites located in the Indian Ocean (Mauritius) and the IAA (Great Barrier Reef, Moorea, Togian and Vanuatu). However, distinguishing between the turnover and PD components of PBD showed that the level of phylogenetic turnover was roughly low for each pairwise comparison (e.g. values of PhyloSor_Turn_ ranging from 0.07 to 0.35, see Table 2). In fact, the high level of PBD found between Panama and the other sites was mostly explained by their difference in PD, as shown by the PhyloSor_PD_ index that ranged from 0.42 to 0.49 (Table 2, Fig. 5a). This result can be explained by the fact that Panama differs greatly from the other sites due to its low level of PD that is directly related to a low species richness (see Table S1). In contrast, the PD component of PBD showed rather low values for each between-site comparison excluding Panama (i.e. values of PhyloSor_PD_ ranged from 0.01 to 0.17, Table 2, Fig. 5b), as these sites displayed comparable levels of PD (i.e. from 11.9 to 17.7, Table S1). These results suggest that using ‘broad-sense’ measures of PBD such as the PhyloSor index may greatly over-estimate the level of spatial turnover of lineages if the two sites show contrasting levels of phylogenetic diversity.

Overall it appears that the IAA lies at the heart of a phylogenetic radiation, with the broader Indo-Pacific exhibiting a high degree of nestedness. In many ways this reflects the taxonomic similarities seen in both fishes and corals across the domain [44], most of the Indo-Pacific being a low diversity subset of the IAA. A marked departure from this pattern was only found in geographically marginal locations of the East Pacific, such as Panama, where a history of isolation and species loss has resulted in an unusual low and unique diversity of coral reef fish species [44]. In addition, the development of a complex mosaic of reef habitats in the IAA during the Oligocene/Miocene has been shown to be a significant driver of cladogenesis in several coral reef fish families such as Labridae [43], hence explaining the high level of PD found in the IAA compared to Panama. Our results suggest that the high levels of PBD between Panama and the other sites is not a result of its historical association with the west Tethys [41] but with a more recent history of isolation and decline [45].

**Figure 6:**
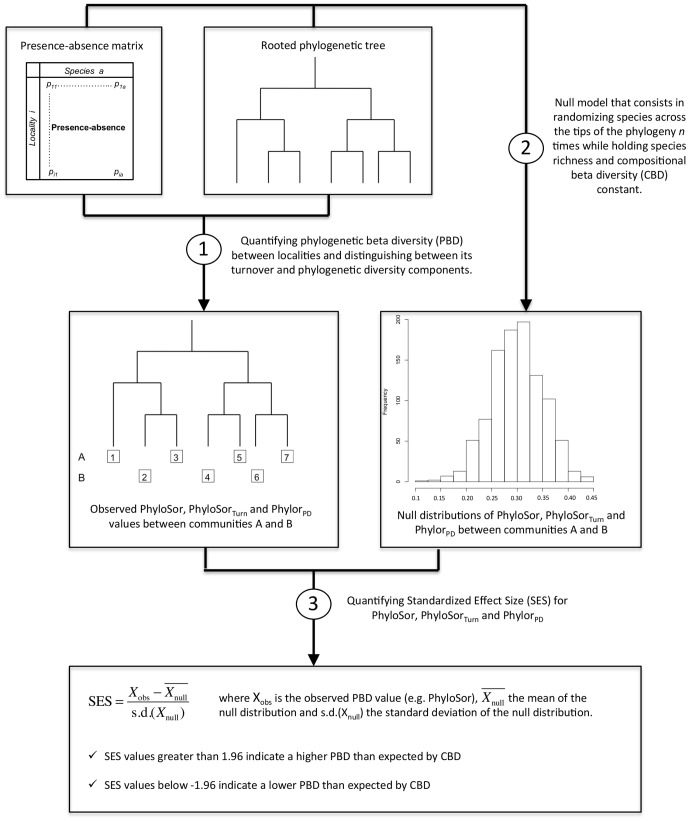
Flow chart of the main steps to quantify standardized effect size of phylogenetic beta diversity.

Recent approaches exploring PBD allowed the differentiation of historical (e.g. speciation and dispersal) vs. niche-based processes (e.g. environmental filtering and niche similarity) in shaping assemblage structure at both local and regional scales [3], [9], [14]. These studies compared observed values of PBD with those obtained by a null model where random assemblages are drawn from the overall species pool (Fig. 6). For instance, if observed values of PBD at the regional scale do not differ from what would be expected by chance alone, phylogenetic structure of regional assemblages is unlikely to be the result of historical processes [14]. In addition, as discussed above, CBD and PBD are highly correlated and an appropriate null model is therefore required to determine whether PBD is higher or lower than expected given CBD. We therefore used a null model approach (see Fig. 6) similar to that applied by Graham *et al.* [3] and Swenson *et al.* [46]. Specifically, a null distribution of PBD values was generated by randomizing species across the tips of the labrid phylogeny 9999 times while holding species richness and CBD constant. A standardized effect size (SES) was then calculated for the PhyloSor index and its turnover and PD components using the mean and standard deviation of the null distribution as follows [47]:
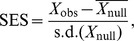
where X_obs_ is the observed PhyloSor value, 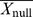 the mean of the null distribution and s.d.(X_null_) the standard deviation of the null distribution. SES values greater than 1.96 indicate a higher PBD than expected by CBD while SES values below -1.96 indicate a lower PBD than expected by CBD. Specifically, we aimed at exploring whether PBD measures that account for differences in PD (PhyloSor) showed similar SES values than PBD measures that do not account for PD differences (PhyloSor_Turn_).

This null model analysis showed non-random patterns of PBD for 6 pairwise comparisons out of 14 (Table 2), particularly those involving the GBR, Moorea, Togian and Vanuatu sites. However, accounting or not for PD differences between sites showed contrasting results for 3 pairwise comparisons (Mauritius-Togian, Moorea-Vanuatu, Togian-Vanuatu). For instance, the PhyloSor index showed a higher PBD than expected given CBD between the Togian and Vanuatu sites (SES = 2.96, see Table 2). This may be interpreted as a non-random spatial turnover of lineages resulting from either past speciation and extinction events or dispersal limitation of lineages or niche-based processes or a combination of both. However, controlling for PD differences between these two sites revealed a random pattern of lineage turnover (SES = 1.49). Conversely, using the PhyloSor index did not show a higher or lower PBD than expected by CBD between the Moorea and Vanuatu regions (SES = 1.04, see Table 2), while the PhyloSor_turn_ index did (SES = 2.05). Such discrepancies suggest that accounting for PD differences between sites may lead to contrasting conclusions in regards to the degree of phylogenetic structure in assemblage composition. Here our aim was not to disentangle the relative roles of ecological and evolutionary processes in shaping large-scale patterns of phylogenetic structure in coral reef fish communities. This would need analysing a larger dataset. Instead, we were interested in showing how the choice of including or not differences in PD to quantify PBD could lead to contrasting results from a null model.

## Concluding Remarks

Integration of phylogenetic information into a community ecology framework has provided new insights into our understanding of the roles of ecological and evolutionary processes in shaping patterns of community structure at local and regional scales [1], [2], [9], [48], [49]. The present study participates to this emerging field of research called “ecophylogenetics" [48].

Overall, our results suggest that PD gradients may distort phylogenetic turnover patterns if the PBD measures (e.g. PhyloSor or Unifrac) incorporate PD differences between localities (or regions). This finding has important implications in the context of hypothesis testing in community ecology and biogeography [9]. For instance, one might test whether large-scale patterns of PBD can be explained by an environmental filtering process (or lineage filtering process), whereby local communities experiencing different environments contain different lineages [50]. However, these localities encompassing various regions may greatly differ in their level of phylogenetic diversity due to regional processes, e.g. regional differences in the amount of time for speciation [51] and/or differential rates of immigration [52]. Using the PhyloSor (or UniFrac) index that incorporates PD differences may hence make it difficult to distinguish between the relative roles of local-scale processes (e.g. environmental filtering) and regional processes (e.g. time for speciation) in shaping large-scale patterns of PBD. When the environmental filtering hypothesis is to be tested, we therefore recommend the use of the PhyloSor (or UniFrac) index in tandem with its ‘true’ phylogenetic turnover component (PhyloSor_turn_), so as to control for the potential confounding effect of PD differences. Decoupling variation in beta diversity from variation in alpha diversity has rapidly emergered as an important step towards a better understanding of the drivers of community structure across latitudinal and altitudinal gradients [53], [54]. From a phylogenetic perspective, our proposed decomposition of PBD into ‘true’ phylogenetic turnover and PD components participates to this emerging biogeographical issue.

Recent studies aimed at determining the statistical independence of several PBD metrics [11], [25], [55]. For instance, Swenson [11] showed that many PBD metrics (e.g. PhyloSor, UniFrac and two nearest neighbor metrics) were highly related, most of them being able to detect basal vs. terminal PBD. As mentioned by Swenson [11], future studies introducing new PBD metrics would show how these metrics actually provide novel information and strengthen the statistical toolkit of the phylogenetic community ecologist. In line with a previous additive partitioning framework of CBD [19], [26], we provided new insights into a specific class of PBD metrics that belongs to the family of phylogenetic diversity-based dissimilarity measures. Specifically, we propose a new phylogenetic turnover metric that is independent of variation in PD between localities (or regions). We hope that our proposed PBD metrics will help future studies to unravel the mechanisms driving large-scale patterns of biodiversity.

## Supporting Information

Figure S1 **Simulated phylogenetic trees obtained by the Yule (a) and PDA (b) models.**
(TIFF)

Figure S2 **Relationships between the PDB values obtained using two different phylogenetic trees (Yule vs. PDA model).**
(TIF)

Table S1 **Values of phylogenetic diversity (calculated as the total sum of branch length) and species richness of coral reef fish species belonging to the family of Labridae for each studied site.**
(DOCX)

File S1 **R code for quantifying phylogenetic beta diversity and its ‘true’ turnover and phylogenetic diversity components (requires R; download R software from** http://cran.r-project.org **).**
(R) Click here for additional data file.

File S2 **R code to run the example illustrated in** **Table 1** **and** **Figure 2** **(requires R; download R software from** http://cran.r-project.org **).**
(R) Click here for additional data file.

File S3 **Species occurrence matrix used in** **Table 1** **and** **Figure 2** **.**
(CSV)

File S4 **Hypothetical phylogenetic tree used in** **Table 1** **and** **Figure 2** **.**
(NWK)
