# Correction: Quantifying Phylogenetic Beta Diversity: Distinguishing between ‘True’ Turnover of Lineages and Phylogenetic Diversity Gradients

**DOI:** 10.1371/annotation/6fe0199e-7916-4bb9-9c9e-b854c5cee029

**Published:** 2012-10-08

**Authors:** Fabien Leprieur, Camille Albouy, Julien De Bortoli, Peter F. Cowman, David R. Bellwood, David Mouillot

The sentence 'We express b, c and a using the phylogenetic diversity index [30], [31] that can be calculated as the total branch length of a phylogenetic tree T that contains all species present in a community ' should read as ' Following Nipperess et al. [15], we express b, c and a using the phylogenetic diversity index [30], [31] that can be calculated as the total branch length of a phylogenetic tree T that contains all species present in a community '.

We expressed the a, b and c components using the formulas described in Nipperess et al. [15] because these authors relevantly proposed a phylogenetic diversity based-framework to calculate the matching/dismatching components of a large number of similarity and dissimilarity measures.

In addition, the authors would like to correct an error in equation 5. The equation 5 as indicated in the present version is wrong as this equation corresponds to the Beta-sim index (equation 2). The correct equation 5 that corresponds to the turnover component of the Jaccard's dissimilarity index is : Beta-jtu = 2min(b,c)/a+2min(b,c).

There are also some corrections necessary under the section "Distinguishing between Phylogenetic Diversity Gradients and Spatial Turnover of Lineages: Formulations":

- In the fourth paragraph, the first sentence should read: Following the formula (2) and (5), we obtained the turnover components of the PhyloSor and UniFrac indices, i.e. PhyloSor_Turn_ and UniFrac_Turn_, respectively:...

- In the fifth paragraph, the second sentence should read: It can be expressed using the formula (3) and by replacing *a*, *b* and *c* by the formula (13), (11) and (12), respectively.

- In the fifth paragraph, the fourth sentence should read: . It can be expressed using the formula (6) and by replacing *a*, *b* and *c* by the formula (13), (11) and (12), respectively.

- In the last parapraph, the first sentence should read: Overall, the above examples (Fig. 1 and 2, Table 1) emphasize that PhyloSor_Turn_ and UniFrac_Turn_ are two 'narrow-sense' measures of PBD (i.e. 'true' measures of phylogenetic turnover) that are independent of total branch length difference between the two compared communities (see Fig. 2 and Table 1). 

