# Supplementary figures and images for: Quantifying Phylogenetic Beta Diversity: Distinguishing between ‘True’ Turnover of Lineages and Phylogenetic Diversity Gradients
Source: PLoS One. 2012 Aug 17;7(8):e42760. doi: 10.1371/journal.pone.0042760 (PMC3422232; doi:10.1371/journal.pone.0042760)

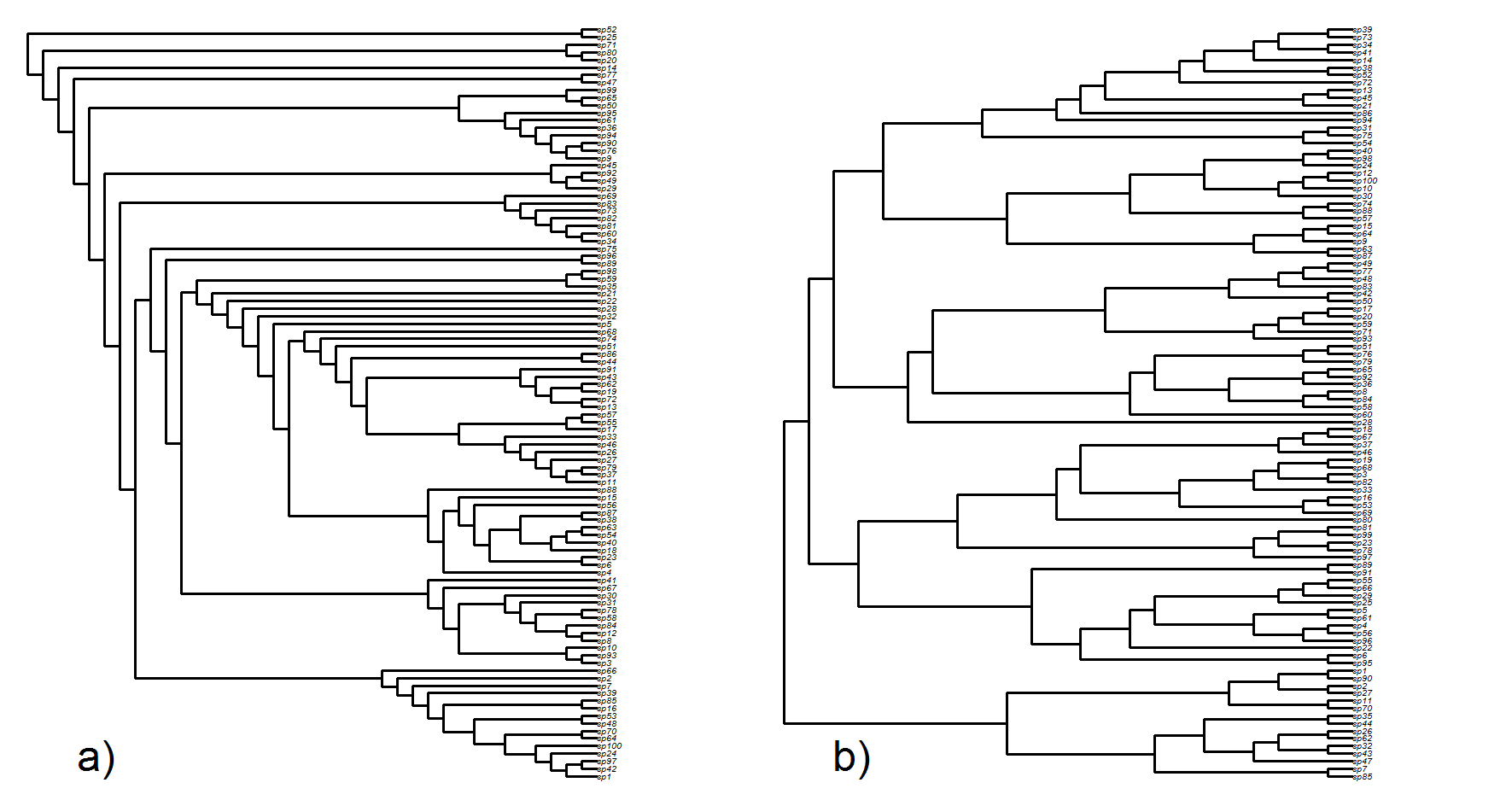

Supplement: Figure S1 — Simulated phylogenetic trees obtained by the Yule (a) and PDA (b) models. (TIFF) [file pone.0042760.s001.tiff]

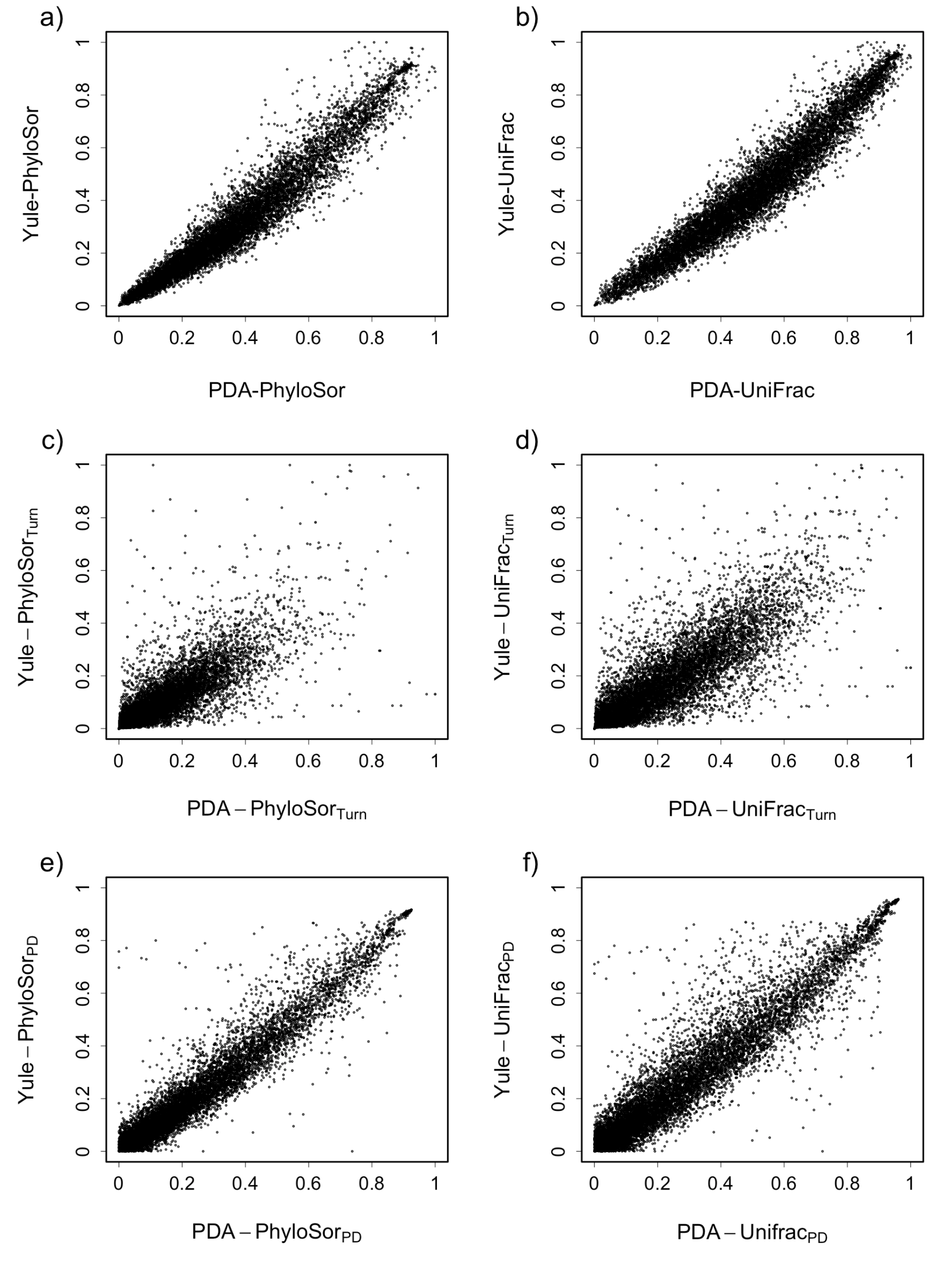

Supplement: Figure S2 — Relationships between the PDB values obtained using two different phylogenetic trees (Yule vs. PDA model). (TIF) [file pone.0042760.s002.tif]
