# Supplementary material for: Quantifying Phylogenetic Beta Diversity: Distinguishing between ‘True’ Turnover of Lineages and Phylogenetic Diversity Gradients
Source: PLoS One. 2012 Aug 17;7(8):e42760. doi: 10.1371/journal.pone.0042760 (PMC3422232; doi:10.1371/journal.pone.0042760)
Supplement: Table S1 — Values of phylogenetic diversity (calculated as the total sum of branch length) and species richness of coral reef fish species belonging to the family of Labridae for each studied site. (DOCX) [file pone.0042760.s003.docx]

**Supplementary Information**

**Table S1.** Values of phylogenetic diversity (calculated as the total sum of branch length) and species richness of coral reef fish species belonging to the family of Labridae for each studied site.

| **Regions** | **Phylogenetic diversity** | **Species richness** |
| --- | --- | --- |
| Mauritius (Indian Ocean) | 11.9 | 27 |
| Togian (Indonesia) | 17.4 | 60 |
| Moorea (Polynesia) | 15.3 | 44 |
| Panama (Eastern Pacific) | 2.8 | 5 |
| Great Barrier Reef (Western Pacific) | 17.7 | 56 |
| Vanuatu (Western Pacific) | 14.4 | 51 |
